# Building From a Stable Foundation? Understanding Stability Skill Proficiency in British Children in the First Year of School

**DOI:** 10.1111/cch.70270

**Published:** 2026-04-01

**Authors:** Michael J. Duncan, Matteo Crotti, Katie Fitton Davies, Eileen Africa, Ricardo Martins

**Affiliations:** ^1^ Centre for Physical Activity, Sport and Exercise Sciences Coventry University Coventry UK; ^2^ Department of Human and Social Science University of Bergamo Bergamo Italy; ^3^ Research Institute for Sport and Exercise Sciences Liverpool John Moores University Liverpool UK; ^4^ Division of Movement Science and Exercise Therapy Stellenbosch University Stellenbosch South Africa; ^5^ Faculty of Kinesiology, Sport and Recreation University of Alberta Edmonton Canada

**Keywords:** balance, fundamental movement skills, motor competence, proficiency

## Abstract

**Objective:**

Stability skill proficiency is purported to be below the norm in children. However, no process‐oriented proficiency data are available examining this issue. This study assessed stability skill proficiency in British children.

**Materials and Methods:**

Stability skill proficiency of 243 (116 boys and 127 girls) English children aged 4–5 years was assessed using the Test of Stability Skills. Stability skill proficiency was classed as proficient, near proficient and poor.

**Results:**

Approximately 60% did not achieve proficiency in any of the three stability skills. Only three children (1.2%) were proficient in all three stability skills. Individual skill proficiency was poor, with proficiency of the roll, rock and back support being 10.3%, 4.9% and 8.2%, respectively.

**Conclusion:**

This study provides preliminary normative data for process‐oriented stability skill in British children aged 4–5 years. As stability skill is a prerequisite for the development of other gross motor skills, such information is important in effective targeting for stability skill intervention.

## Summary

- Stability skills are a key, but often overlooked, aspect of fundamental movement skills, with the predominant approach in the literature focusing on locomotor and object control skills.
- Although some product‐oriented approaches to motor competence assessment include balance subscales, to date, no studies have reported process‐oriented data relating to stability skills in children aged 4–5 years.
- This study presents preliminary process‐based data on stability skill, using the Test of Stability Skills, for Reception‐aged children living in high deprivation in England. The results suggest that stability skill proficiency is poor, at approximately 10%, 5% and 8% for the roll, rock and back support skills, respectively.
- Future work may want to document stability skill proficiency in a more representative sample of children in England to verify the claims of low proficiency suggested in the current work.

## 1 Introduction

Considerable research has examined children's fundamental movement skills (FMS) and their importance in relation to children's development (Lopes et al. 2020; Logan et al. 2015; Robinson et al. 2015). The development of these FMS, involving skills such as running, jumping, throwing and catching, is an important enabler of children's health, enhancing physical activity (Newell 2020), well‐being and academic achievement (Lopes et al. 2020, Logan et al. 2015, Robinson et al. 2015). However, during the processes of child development and maturation, FMS do not develop naturally (Hardy et al. 2010). For FMS proficiency, developmentally appropriate activities are needed. This might, for example, come from motor development activities (with feedback) within physical education (PE) classes in school (Logan et al. 2015).

Consequently, developing proficiency in FMS features as a key component in school PE curricula across the world (Australian Curriculum, Assessment and Reporting Authority 2012; Department of Education 2013; Society of Health and Physical Educators SHAPE America 2013). In England, as one example, the National Curriculum for PE for Key Stage 1 (age 5–7 years) identifies FMS development as a key outcome (Department of Education 2013) and the Early Years Foundation Stage (2–4 years, EYFS; Department of Education 2021). Successive expert statements on behalf of the International Motor Development Research Consortium have, however, identified that the level of FMS proficiency is below levels for children in the United Kingdom and Ireland (Duncan et al. 2022) and children aged 3–5 years (Martins et al. 2024), respectively. However, the predominant approach in the literature on FMS development to date has tended to focus on FMS as comprising only locomotor and object control skills, while failing to fully consider stability skills (Newell 2020; Rudd et al. 2015). Stability skills refer to the body's ability to maintain balance despite the effect of an internal or external force (Newell 2020) and are considered the most basic of FMS (Gallahue et al. 2012). Stability skills and their development are precursors for the development of other FMS (Newell 2020).

It is therefore surprising that stability skills are the least examined category of FMS skills (Rudd et al. 2015; Fitton Davies et al. 2024). There have been suggestions that children's stability skills are low and below expected levels (Rudd et al. 2015), alongside calls for research to understand children's proficiency in stability skills (Rudd et al. 2015; Rudd et al. 2017). In the context of motor assessment, a number of validated test batteries including the Movement Assessment Battery for Children (M‐ABC), Körperkoordinationtest für Kinder (KTK) and the Bruininks–Oseretsky Test of Motor Proficiency (BOTMP) include subscales that provide a score for ‘balance’. Although such information is useful, these aforementioned assessments are all considered product measures of motor competence and are not solely considered a stability skill assessment. To date, there is only one process‐oriented measure of stability skills, the Rudd et al. (2015) Test of Stability Skills (TSS), which was originally validated in children aged 6–10 years but has subsequently been employed in children from the age of 4 onwards (Fitton Davies et al. 2024). Product measures focus on the outcome of movement, where process‐oriented assessments assess how the movement is performed, thereby providing information on movement quality (Lorenzo‐Martínez et al. 2025). Such information is regarded as particularly valuable for providing qualitative insights into children's movement skills and, compared to product‐oriented assessments, is often more sensitive to subtle improvements in motor skills, particularly in young children (Silva 2025). In addition, because process‐oriented measures assess how the movement is performed and the components within the movement, process‐oriented measures are considered superior to product‐oriented measures for designing targeted interventions and informing instruction and feedback to improve motor competence (Bardid et al. 2019).

Yet, to date, there remains a general lack of research specifically examining stability skills in young children, and there are no data presenting children's proficiency in stability skills using process‐oriented measures of stability skill (Fitton Davies et al. 2024; Rudd et al. 2017).

This study therefore presents data on stability skill proficiency of English children in their first year of primary school.

## 2 Methods

### 2.1 Participants

Following institutional ethics approval and written parental informed consent, 243 children aged 4–5 years (85% Caucasian; 116 boys and 127 girls; mean = 4.2 years; standard deviation [SD] = 0.4) in Reception classes from six central England primary schools participated in this study. Convenience sampling was used to select schools. All schools involved were located in deprived areas (within the top third most deprived areas) within England (Index of Multiple Deprivation, Public Health England 2019).

### 2.2 Assessment of Stability Skills

The TSS (Rudd et al. 2015) was employed to assess stability skills. The TSS is a process‐oriented assessment comprising three skills: the log roll, the rock and the back support. The skills within the TSS have been fully explained previously (see Rudd et al. 2015) and feature multiple aspects that are recommended to be taught in curriculum gymnastics sessions in Reception PE classes, making the TSS relevant for Reception‐aged children in England. In brief, the TSS comprises performance of the following skills:

Log roll: The log roll assesses the ability to maintain postural control and body orientation while moving. The child lies on a gymnastic mat and rolls sideways with arms and legs extended and slightly elevated from the ground. The log roll requires the child to maintain a straight body line, with extended arms and legs throughout the roll, without touching the ground with hands or feet for four rotations of the body.

Rock: The rock assesses body orientation, weight transfer and control. The child starts in a seated rocking position and rocks back and forth twice on their lower back, shoulders and neck in a manner similar to learning a front roll. The rock involves maintaining a curled position during the rocking movement and then transitioning to a standing position at the end of the movement.

Back support: The back support assesses whole‐body postural control and stability. The child holds a static wedge shape with their body, commonly known as a support position, maintaining a straight body position with arms straight and together, supporting their weight on their hands/elbows and heels for a period of 45 s.

According to Rudd et al. (2015), the three tests within the TSS provide an effective way to examine a child's ability to orient and stabilise their bodies in space within a field setting. The rock and log roll assess both orientation and stability, whereas the back support mainly assesses stability and torso strength, with confirmatory factor analysis identifying the skills fitting into one latent construct for ‘stability’ (Rudd et al. 2015).

Children were filmed performing the skills (Sony Handicam FDR‐AX43). Skills were subsequently scored following the guidelines provided by Rudd et al. (2015). Skills within the TSS comprise several behavioural components that are scored 0 (not present) or 1 (present). Skills are summed to provide a total score for each skill, reflecting a child's overall stability skill. Total scores range from 0 (no criteria are present across trials) to 24 (all criteria are present across two trials).

Administration of the TSS followed recommended guidelines (Rudd et al. 2015). Children watched one demonstration of each skill provided by a trained researcher prior to undertaking one practice trial, before completing two recorded trials. Recorded trials were scored by a trained researcher who had previously undertaken training in scoring the TSS alongside a gold‐standard scorer. Inter‐ and intra‐rater reliability analyses were performed for the TSS. Intra‐class correlation coefficients for inter‐ and intra‐rater reliability were 0.905 and 0.957. Recognising that the original validation of the TSS was undertaken for children aged 6–10 years, prior to administration, we compared scores on the TSS with scores on the stability subscales (sum of *z*‐scores for lateral jumping and sideways platforms) of the Motor Competence Assessment (Rodrigues et al. 2022) in a subsample of forty‐six 4–5‐year‐olds (20 boys and 26 girls; mean age ± SD = 4.2 ± 0.4 years). Pearson product‐moment correlation for the relationship between TSS scores and stability subscale scores was *r* = 0.606, *p* = 0.001, indicating that children who scored highly in the TSS also scored highly on the stability subscale of the MCA, providing preliminary evidence of validity of the TSS in 4–5‐year‐old children.

### 2.3 Statistical Analysis

Descriptive statistics and frequencies for each of the skills within the TSS (the log roll, the rock and the back support) were calculated. For each of the skills within the TSS and using previously established procedures (Duncan et al. 2020; O'Brien et al. 2016), ‘proficiency’ was defined as correct performance of all skill components on both trials, and ‘near proficiency’ was defined as correct performance of all components but one on both trials. Classification as ‘poor’ was for any score below these two categories (i.e., if the performance was incorrect in two or more of the components on both trials). The percentage of children classified as proficiency, near proficiency and poor in each of the three skills was determined. Independent *t*‐tests were subsequently performed to examine any sex differences in the skills within the TSS. The Statistical Package for the Social Sciences (SPSS, version 28) was used for all analyses.

## 3 Results

**FIGURE 1:**
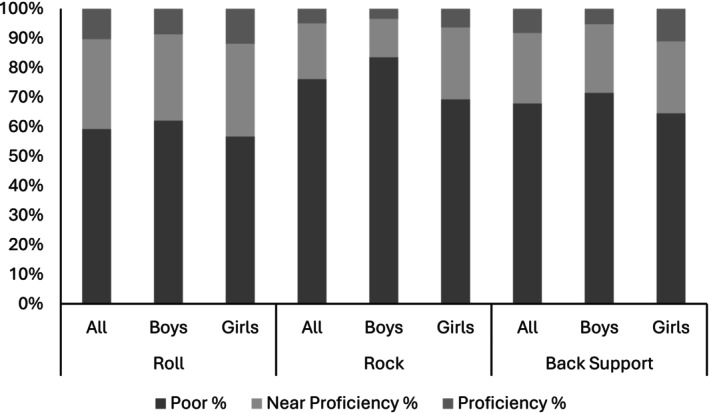
The percentage of the whole sample, boys and girls classed as ‘poor’, ‘near proficiency’ and ‘proficiency’ in roll, rock and back support stability skills.

For the whole sample, approximately 60% (*n* = 143) did not achieve proficiency in any of the three stability skills. Only three children (1.2%) achieved ‘proficiency’ in all three of the stability skills. The proportion of the whole sample, and of boys and girls, who achieved proficiency in the rock, roll and back stability skills is presented in Figure 1.

In regard to the individual skills, the poorest performance was for the rock, where 79.1% of boys and 69.3% of girls were rated as ‘poor’. This was mirrored for the roll, where 62.1% of boys and 56.7% of girls were rated as ‘poor’, and for the back support, where 71.5% of boys and 64.6% of girls were rated as ‘poor’.

Results from independent *t*‐tests examining sex differences in the three stability skills indicated no significant differences in the roll (*t* = −1.219, df = 241, *p* = 0.224, *d* = 1.67) or the back support (*t* = −0.721, df = 241, *p* = 0.472, *d* = 2.77). There was a significant difference in scores on the rock (*t* = −2.387, df = 241, *p* = 0.018, *d* = 2.12) in favour of the girls. Mean ± SD and 95% confidence intervals for the roll, rock and back support are presented in Table 1.

**TABLE 1:** Mean ± SD and 95% confidence intervals for the roll, rock and back support stability skills.

	Boys			Girls		
	Mean	SD	95% CIs	Mean	SD	95% CIs
Roll (0–6)	2.8	1.6	2.6–3.2	3.1	1.7	2.8–3.4
Rock(0–8)	3.4	1.9	3.1–3.8	4.1	2.0	3.7–4.5
Back support (0–10)	4.5	2.5	4.5–5.4	5.2	2.9	4.6–5.7

## 4 Discussion

This study examined stability skill proficiency in English children aged 4–5 years. Overall, proficiency was low in this sample, and girls only significantly outperformed the boys on ‘the rock’ within the Rudd TSS (Rudd et al. 2015). The EYFS and National Curriculum for PE in England (Department of Education 2013) emphasise the importance of children developing FMS as a foundation for physical activity and sports participation. To date, no study has documented the proficiency level of children in stability skills using process‐oriented measures. This is surprising given the underpinning role that stability skills play in the development of FMS and that process‐oriented information, describing the quality of movement rather than the outcome, is particularly useful when attempting to understand motor skill development in younger children (Lorenzo‐Martínez et al. 2025). This is because such information allows understanding of which components of movement may need addressing to result in overall movement quality improvement (Silva 2025). Although a number of motor competence assessments include subscales that assess ‘balance’ including the KTK, M‐ABC and BOTMP, these measures are product‐based assessments. It is also important to note that there is existing normative data relating to balance performance for UK children of similar ages, particularly for the M‐ABC (Henderson and Barnett 2023). The current study does not replicate this aforementioned research but instead presents preliminary process‐based data on stability skill for Reception‐aged children in England. The results of the present study highlight that stability skill proficiency is poor, with proficiency at approximately 10%, 5% and 8% for the roll, rock and back support, respectively.

Understanding proficiency levels of stability skills is a necessary first step for early years professionals and teachers to understand where their pupils might ‘sit’ in terms of their physical development within the EYFS and the subsequent development of FMS within the school curriculum targets. Examining skill proficiency and which skills are harder to master can enable teachers and researchers to identify areas of motor skill deficiency. This information can subsequently be used to implement strategies to facilitate children's motor development relating to FMS in the PE curriculum. It is, however, important to note that the TSS, used in the current study, was originally validated for children aged 6–10 years. No process‐based measure of stability skills has been validated for use in children aged 4–5 years, hence our decision to employ the TSS. Comparison of scores on the TSS with scores on the stability subscale for the MCA, a product measure of motor competence, in 4–5‐year‐olds, revealed a positive correlation (*r* = 0.606), providing some initial evidence of validity of the TSS in this population. Despite this, it is possible that the low proficiency scores reported in the current study may be a result of floor effects in using the TSS in 4–5‐year‐olds, potentially overstating poor performance. This age was selected to provide an indication of stability skill proficiency for children at the age where such skills are suggested to be mastered (Gallahue et al. 2012). However, future work is needed that fully explores the psychometrics of the TSS in 4–5‐year‐olds beyond the initial evidence presented in the current study. A future comparison of product‐oriented motor assessments that include a balance subscale or component (e.g., the KTK or M‐ABC) with the TSS would also be useful in exploring the agreement between different measures of stability skill in this age group of children.

We are conscious that the current study focused on a convenience sample of children in England from low SES backgrounds. The use of this sample may also, in part, explain the lower levels of proficiency we report, particularly because global data on motor competence in children, including children from England, have consistently indicated lower levels of motor competence in children from low SES backgrounds (Bolger et al. 2021). Future work may want to document stability skill proficiency in a more representative sample of children in England to verify the claims of low proficiency suggested in the current work. However, the present study provides the first empirical data on process‐oriented stability skills in English 4–5‐year‐olds and suggests that such skills are in need of further development.

## Author Contributions

**Michael J. Duncan:** conceptualization, methodology, investigation, formal analysis, writing – original draft. **Matteo Crotti:** investigation, writing – review and editing. **Katie Fitton Davies:** conceptualization, methodology, writing – review and editing. **Eileen Africa:** conceptualization, writing – review and editing. **Ricardo Martins:** methodology, investigation, formal analysis, writing – review and editing.

## Funding

The authors have nothing to report.

## Ethics Statement

This research was approved by the Ethics Committee of Coventry University (Ref: P125525).

## Conflicts of Interest

The authors declare no conflicts of interest.

## Data Availability

Data from the study are available from the corresponding author upon reasonable request.
